# Mental health of young people amidst COVID-19 pandemic in Bangladesh

**DOI:** 10.1016/j.heliyon.2021.e07173

**Published:** 2021-05-28

**Authors:** Md. Abdullah Saeed Khan, Sourav Debnath, Md. Shahnoor Islam, Susmita Zaman, Noor-E- Ambia, Anindita Das Barshan, Mohammad Sorowar Hossain, Tamanna Tabassum, Monjur Rahman, Mohammad Jahid Hasan

**Affiliations:** a Pi Research Consultancy Center, Dhaka, Bangladesh; b Dhaka Medical College Hospital, Dhaka, Bangladesh; c Biomedical Research Foundation, Dhaka, Bangladesh

**Keywords:** COVID-19, Anxiety, Depression, Stress, GAD-7, PHQ-9, Lockdown, Not diagnosed

## Abstract

**Background:**

The psychological burden of the coronavirus disease 2019 (COVID-19) outbreak and lockdown strategy among young people not diagnosed with COVID-19 in the general population remains unknown and often have been overlooked. The objective of the study was to assess the prevalence and predictors of anxiety, depression and stress among young people diagnosed with COVID-19 of Bangladesh amidst the pandemic.

**Methods:**

A cross-sectional online survey was conducted from 1 May to 30 May 2020 using an online Google form-based questionnaire posted on Facebook. A snowball sampling approach was used for data collection. A total of 974 self-declared healthy individuals not diagnosed with COVID-19 participated here. Anxiety, depression and stress were measured using Bangla validated Generalized Anxiety Disorder Scale-7 (GAD-7), Patient Health Questionnaire (PHQ-9) scale, and Perceived Stress Scale (PSS), respectively. Statistical software SPSS 20 was used for analysis.

**Result:**

Average age of the population was 25.86 ± 6.26 (SD) years with nearly half (48.6%) of them being young people (15 to ≤24 years). Most of the participants were male (76.3%). The overall prevalence of anxiety, depression and stress was found to be 64.1%, 73.3% and 69.4%, respectively. Young people had significantly higher proportion of anxiety (67.2% vs 61.1%), and depression (78.2% vs 68.7%) compared to adults (p = 0.045 and p < 0.001, respectively). However, most of the participants had mild depression (30.3%), minimal anxiety (31.4%), and moderate stress (67.5%), and severity of depression and anxiety was higher in the young participants. The mean GAD-7, PHQ-9 and PSS scores were 7.57 ± 5.61, 9.19 ± 6.15 and 16.02 ± 5.55 (SD), respectively. On multivariable logistic analysis, unemployment (Adjusted Odds Ratio [AOR] 3.642; Confidence Interval [CI]: 1.005–13.200; p < 0.05) was the single most important predictor of depression. For stress, unemployment (AOR 1.399; CI: 1.055–1.855), and female sex (AOR 1.638; CI: 1.158–2.317) were significant predictors.

**Conclusion:**

Anxiety, depression and stress were highly prevalent among young people (≤24 years) not diagnosed with COVID-19 in Bangladesh amidst the pandemic. Unemployment is the most common underlying determinant. Authorities should address the issue on a priority basis.

## 1 Introduction

Coronavirus disease-2019 (COVID-19) originated in Wuhan city, China, in December 2019 [1] and spread quickly to other countries [2]. The World Health Organization (WHO) declared COVID-19 a public health emergency of international concern (PHEIC) on 30th January 2020 and announced it as a pandemic on 11^th^ March [2, 3]. By this time, the health sector of almost all countries activated themselves with their highest level of capacity. Governments launched existing and innovative strategies to combat COVID-19. Public health authorities prioritized preventive strategies to limit the spread of the disease [4]. As COVID-19 had already been marked as a highly contagious disease, mostly spread via respiratory droplets, by direct contact with infected persons, or by contact with contaminated objects and surfaces, social distancing became the mainstay of prevention [5]. Social distancing is a very new term for a certain population, and there is no easy way to make people accustomed to it in a short period of time. Thus, the lockdown system was adopted by many countries of the world [6], where people were confined within a defined area, and more strictly, people were not allowed to go outside. Although the process of lockdown is beneficial in terms of infection reduction, it has severely affected the economy from the individual to global levels [7, 8]. People from all levels of status were affected either by the disease or due to its socioeconomic consequences [9].

COVID-19 has become a concern in developing countries such as Bangladesh, which is densely populated with a struggling health care system. People were afraid because of inadequate protective and management capacity. Additionally, they were experiencing economic loss due to drastic lockdown measures [10]. Moreover, evidence suggests that individuals who are kept in isolation and quarantine experience significant distress in the form of anxiety, anger, confusion and posttraumatic stress symptoms [11]. Anxiety and concerns in society globally affect every individual in various aspects. Nonetheless, uninfected individuals are expected to have a mild impact compared to infected individuals. However, the ‘infodemic’ caused by electronic and social media in the form of rumors and misinformation might have made reactions worse among the former group [12].

Mental health is often an ignored issue in countries such as Bangladesh [13]. Earlier studies carried out among the adult population of Bangladesh suggested a high prevalence of symptoms of anxiety, depression and stress as well as the considerable presence of suicidal ideation among a certain population [14, 15]. The initial progression of events indicated that people, particularly who were young, considered the lockdown steps lightly [16]. This indicates a difference in reactions of uninfected persons to the pandemic than those who were infected. To date, very few studies have exclusively addressed the mental health of persons who were not diagnosed with COVID-19. Considering the relevance of all the above factors, the objective of the study was to assess the prevalence and predictors of anxiety, depression and stress among the young adults not diagnosed with COVID-19 in Bangladesh amidst the pandemic.

## 2 Materials and methods

### 2.1 Study period, design and study subjects

The study was conducted from 1st May to 30th May 2020. As the number of cases was increasing in March, the Government of the People's Republic of Bangladesh adopted country-wide lockdown as a public health measure to mitigate the transmission of the disease [17]. Hence, health center-based or community-based surveys were not practicable to perform, and face-to-face interviews were not feasible as well. Therefore, we conducted a Facebook-based online survey among the general population not diagnosed with COVID-19 living in Bangladesh. People aged more than and equal to 15 years, not infected by COVID-19, and having no diagnosed psychiatric illness with or without treatment using Facebook with minimum proficiency to Bangla to answer the questions of the current study online were primarily targeted. A Google form was created and circulated on Facebook (mostly used social media in Bangladesh). Online informed written consent was obtained from all the participants before they answered the questions.

### 2.2 Sampling technique and study sample

The actual prevalence of anxiety, depression or stress disorder in the general population has not yet been estimated. Therefore, considering a 50% prevalence with a 95% CI and agreeing 5% error, the total sample size was estimated to be 384. In this survey, a total of 1038 responses were received, and among them, 1031 agreed to participate and completed the questionnaire. Additional 57 responses were excluded due to incomplete information. Excluding all, a total of 974 responses were considered for final analysis.

### 2.3 Development of study tool

The research tool was prepared based on the existing literature available on the mental health of a population in isolation. The Google Form is used for designing and developing web-based questionnaires that are automatically hosted via a unique URL. A Facebook post was made with details of the aim and objectives, study procedure, and consent statement and further promoted with an unique URL generated for the questionnaire of this study. This URL link gave people round the clock access from anywhere in Bangladesh. The responses were secured using the “Cloud” database (Google Drive), where the data were automatically sorted, scaled and scored by custom Excel formulae. The questionnaire had three parts: a. consent statement, b. demographic details of the participants and c. questions for assessment of anxiety, depression and stress.

### 2.4 Measurement of anxiety, depression and stress

Three well-known scales, the Generalized Anxiety Disorder 7-item (GAD-7) scale [18], Patient Health Questionnaire (PHQ-9) [19] and Perceived Stress Scale (PSS), were used to assess the anxiety, depression and stress of the respondents [20]. We used the validated Bengali versions of the questionnaires [21, 22, 23]. All these scales showed acceptable reliability among our participants (Cronbach's Alpha was 0.858, 0.794 and 0.630 respectively for GAD-7, PHQ-9 and PSS scales, respectively).

The severity of anxiety was measured by the generalized anxiety disorder-7 (GAD-7) scale. The response options were as follows: 0 = “not at all”, 1 = “several days”, 2 = “more than half the days”, and 3 = “nearly every day” for two weeks. The total score ranged from 0 to 21, with a higher score indicating severe anxiety disorder. For the GAD-7, a total score of 0–4 indicates minimal anxiety, 5–9 indicates mild anxiety, 10–14 indicates moderate anxiety and 15–22 indicates severe anxiety.

Depression was measured using the PHQ-9 based on the diagnostic criteria for depression from the Diagnostic and Statistical Manual of Mental Disorders, 4th Edition (DSM-IV). This is an independent structured mental health professional (MHP) interview including 9 depression modules from the full PHQ. The response options were like that of GAD-7 scale: 0 = “not at all”, 1 = “several days”, 2 = “more than half the days” and 3 = “nearly every day”. A two-week recall period was used. The total score ranged from 0 to 27, where depression severity was characterized as ‘none’ if the score was 0–4, mild if 5–9, moderate if 10–14, moderately severe if 15–19 and severe in cases 20–27.

The Perceived Stress Scale (PSS) is a 14-tool containing structured measure designed to determine “the degree to which situations in one's life are appraised as stressful.” Here response categories were: 0 = “never”, 1 = “almost never”, 2 = “sometimes”, 3 = “fairly often”, 4 = “very often”, and two-week recall period was used. Individual scores on the PSS can range from 0 to 40. Higher scores indicating higher perceived stress. Scores ranging from 0 to 13 are considered low stress. The moderate stress ranges from 14-26, and 27–40 is considered high.

### 2.5 Outcome definitions

For predictor outcome relationship assessment outcome were defined as follows. Anxiety was considered present when the score in GAD-7 scale was ≥5 (mild to severe anxiety). Depression was considered present when the score in PHQ-9 scale was ≥5 (mild to severe depression). Finally, stress was considered present when the score in PSS scale was ≥14 (moderate to high stress). A score below these cut-off points were deemed as absence of the corresponding mental health problem.

### 2.6 Predictors of anxiety, depression and stress

Demographic variables recorded at baseline were considered candidates for predictors of outcome. The variables included were age, sex, residence, education, occupation, employment status (if employed), business status (if doing business), being health care worker or not, and monthly income.

### 2.7 Data cleaning and analyses

Due to automation of the Google form, filled data were recorded into the Google drive as sheets in ‘comma separate value’ (csv) format. The sheet was organized and imported into Microsoft Excel and subsequently into the statistical software SPSS 20 (SPSS Inc, Chicago, IL, USA) for final analysis. Exploratory data analysis was carried out to describe the study population, where categorical variables were summarized using frequency tables and continuous variables were summarized using measures of central tendency and dispersion (mean, median and standard deviation.) Bivariate analysis was used to assess the predictors of underlying anxiety, depression and stress. Univariate and multivariate logistic regression models were built to see the association of predictor variables with outcome. After initial screening of predictors in the univariate models, only statistically significant variables were included in the final multivariate logistic regression model and these selected variables were forced into the model using enter method. In all cases, the level of significance was a *p*-value <0.05.

### 2.8 Ethics statement

Before the commencement of the study, formal ethical approval was obtained from the Ethical Review Committee (ERC) of the Biomedical Research Foundation (BRF) (Memo no: BRF/ERB/2020/003). Bangladesh. All participants gave informed written consent before participation on the initial part of the online questionnaire form.

## 3 Results

A total of 974 individuals were included in the final analysis. The mean age of the participants was 25.86 ± 6.26 years (SD) and nearly half of the respondents (n = 473, 48.6%) were young (≤24 years). Most of the participants were male (n = 743, 76.3%) with statistically similar distribution between young adults (≤24 years) and adults (>24 years) (p = 0.184). Majority were single in ≤24 years group (n = 441, 93.2%), and married in >24 years group (n = 261, 52.1%, p < 0.001). More than three-quarter participants (n = 749, 77%) were from urban area with a significantly higher proportion of them being adults (p < 0.001). Half (n = 487, 50.0%) of the participants were students, and nearly three-fourths (n = 712, 73.1%) completed their graduation and postgraduate education. Majority of the students were young people (n = 406, n = 85.8%, p < 0.001). Among all participants, 106 (10.9%) were health care workers. Of all, 495 (56.2%) participants had monthly income ≤30000 BDT. Approximately 59 (6.1%) of the participants lost their job, and 73 (7.5%) had either closed their business or lost their investments during lockdown. The predominant source of information about COVID-19 was Facebook (n = 627, 64.4%). Details are summarized in Table 1.

**Table 1:** Characteristics of the study population (n = 974).

Characteristics	Total	≤24 years	>24 years	*p*-value
	n (%)	n (%)	n (%)	
n (%)	974 (100)	473 (48.6)	501 (51.4)	
Age (years), mean ± SD	25.86 ± 6.26	21.34 ± 2.13	30.13 ± 5.87	<0.001
Sex				
Male	743 (76.3)	352 (74.4)	391 (78.0)	0.184
Female	231 (23.7)	121 (25.6)	110 (22.0)	
Marital Status				
Married	291 (29.9)	30 (6.3)	261 (52.1)	<0.001
Single	683 (70.1)	441 (93.2)	234 (46.7)	
Residence				
Urban	749 (77.0)	326 (68.9)	422 (84.6)	<0.001
Rural	224 (23.0)	147 (31.1)	77 (15.4)	
Education				
SSC and below SSC	58 (6.0)	49 (10.4)	9 (1.8)	<0.001
HSC and undergraduate	204 (20.9)	172 (36.4)	32 (6.4)	
Graduate and above	712 (73.1)	252 (53.3)	460 (91.8)	
Occupation				
Government job	70 (7.2)	2 (0.4)	68 (13.6)	<0.001
Non-government job	178 (18.3)	26 (5.5)	152 (30.3)	
Housewife	29 (3.0)	3 (0.6)	26 (5.2)	
Business	42 (4.3)	5 (1.1)	37 (7.4)	
Student	487 (50.0)	406 (85.8)	81 (16.2)	
Unemployed	138 (14.2)	27 (5.7)	111 (22.2)	
Others^a^	30 (3.1)	4 (0.8)	26 (5.2)	
Health care worker				
Yes	106 (10.9)	16 (3.4)	90 (18.0)	<0.001
No	868 (89.1)	457 (96.6)	411 (82.0)	
Monthly Income (BDT)				
≤30000	495 (56.2)	278 (68.0)	217 (46.0)	<0.001
>30000	386 (43.8)	131 (32.0)	255 (54.0)	
Job Status				
Full time job	196 (20.1)	14 (3.0)	182 (36.3)	<0.001
Part time job	53 (5.4)	24 (5.1)	29 (5.8)	
Lost job due to lockdown	59 (6.1)	24 (5.1)	35 (7.0)	
Not applicable	666 (68.4)	411 (86.9)	255 (50.9)	
Business status				
Ongoing business	31 (3.2)	7 (1.5)	24 (4.8)	0.001
Business closed for now	64 (6.6)	22 (4.7)	42 (8.4)	
Lost investment	9 (0.9)	3 (0.6)	6 (1.2)	
Not applicable	870 (89.3)	411 (93.2)	429 (85.6)	
Source of information regarding COVID-19				
Facebook	627 (64.4)	301 (63.6)	326 (65.1)	0.641
Other media	347 (35.6)	172 (36.4)	175 (34.9)	

*p*-value determined by Independent samples *t* test and Chi-square test where appropriate.

a Other jobs include-intern, house tutor, day laborer, filmmaker, painter, freelancer, and self-employed physician.

**Figure 1:**
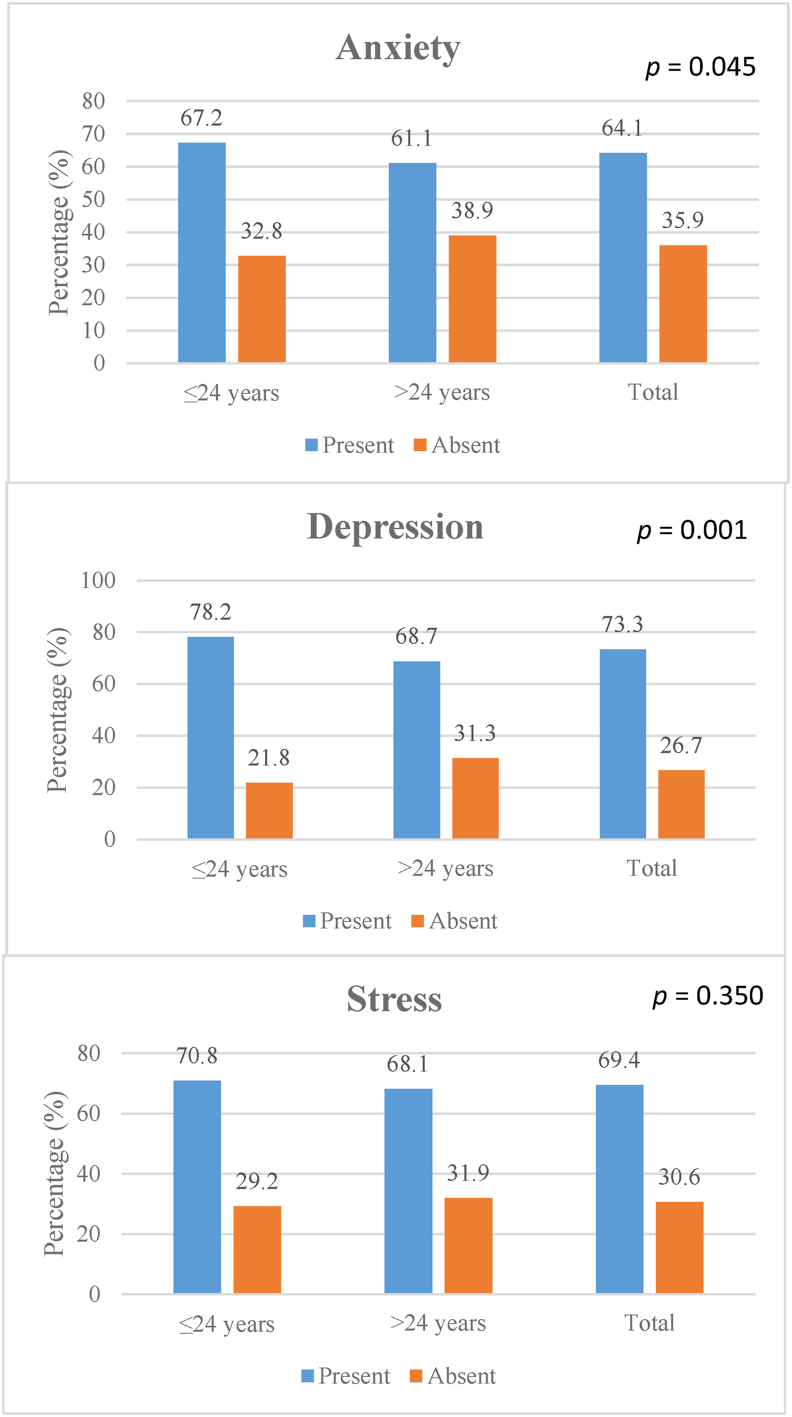
Prevalence of anxiety, depression and stress among the participants (n = 974). Anxiety was defined as having a GAD-7 scale score ≥5 (mild to severe anxiety). Depression was defined as having a PHQ-9 scale score ≥5 (mild to severe depression). Stress was defined as having a PSS scale score ≥14 (moderate to high stress). A score below these cut-off points were considered negative for corresponding scale. Age ≤24 years was considered young adult and >24 years was considered adult. *p*-value was determined by Chi-square test.

The overall prevalence rates of anxiety, depression and stress were 4.1%, 73.3% and 69.4% respectively (Figure 1). Young adults had significantly higher proportion of anxiety (67.2% vs 61.1%), and depression (78.2% vs 68.7%) than adults (p = 0.045 and p < 0.001, respectively). However, prevalence of stress (70.8% vs 68.1%) was statistically similar between those groups (p = 0.350) (Table 2). Female participants had significantly higher proportion of anxiety (76.6% vs 60.2%), depression (82.7% vs 70.4%) and stress (66.9% vs 77.5%) than male (p < 0.001, p < 0.001 and p = 0.002, respectively). Depression was significantly more common in single participants than married ones (76.4% vs 66.0%, p = 0.001). Unemployed participants had significantly higher proportion of depression and stress than others (p = 0.001 and p = 0.034, respectively). Those who lost job during lockdown were significantly more likely to have anxiety and depression than others (p = 0.043 and p = 0.008, respectively). No impact of information source on anxiety, depression and stress was noted. See Table 2 for details.

**Table 2:** Participant characteristics in relation to anxiety, depression and stress (n = 974).

Characteristics	Anxiety		p-value	Depression		p-value	Stress		p-value
	Present	Absent		Present	Absent		Present	Absent	
n (%)	624 (64.1)	350 (35.9)		714 (73.3)	260 (26.7)		676 (69.4)	298 (30.6)	
Age (years), n (%)									
≤24	318 (67.2)	155 (32.8)	0.045	370 (78.2)	103 (21.8)	0.001	335 (70.8)	138 (29.2)	0.350
>24	306 (61.1)	195 (38.9)		344 (68.7)	157 (31.3)		341 (68.1)	160 (31.9)	
Sex, n (%)									
Male	447 (60.2)	296 (39.8)	<0.001	523 (70.4)	220 (29.6)	<0.001	497 (66.9)	246 (33.1)	0.002
Female	177 (76.6)	54 (23.4)		191 (82.7)	40 (17.3)		179 (77.5)	52 (22.5)	
Marital Status, n (%)									
Married	177 (60.8)	114 (39.2)	0.169	192 (66.0)	99 (34.0)	0.001	191 (65.6)	100 (34.4)	0.096
Single	447 (65.4)	236 (34.6)		522 (76.4)	161 (23.6)		485 (71.0)	198 (29.0)	
Residence, n (%)									
Urban	485 (64.8)	264 (35.2)	0.460	559 (74.6)	190 (25.4)	0.106	523 (69.8)	226 (30.2)	0.575
Rural	139 (62.1)	85 (37.9)		155 (69.2)	69 (30.8)		152 (67.9)	72 (32.1)	
Education, n (%)									
SSC and below SSC	37 (63.8)	21 (36.2)	0.778	48 (82.8)	10 (17.2)	0.119	37 (63.8)	21 (36.2)	0.585
HSC and undergraduate	135 (66.2)	69 (33.8)		155 (76.0)	49 (24.0)		140 (68.6)	64 (31.4)	
Graduate and above	452 (63.5)	260 (36.5)		511 (71.8)	201 (28.2)		499 (70.1)	213 (29.9)	
Occupation, n (%)									
Government job	37 (52.9)	33 (47.1)	0.073	47 (67.1)	23 (32.9)	0.001	41 (58.6)	29 (41.4)	0.034
Non-government job	104 (58.4)	74 (41.6)		113 (63.5)	65 (36.5)		113 (63.5)	65 (36.5)	
Housewife	22 (75.9)	7 (24.1)		24 (82.8)	5 (17.2)		22 (75.9)	7 (24.1)	
Business	26 (61.9)	16 (38.1)		25 (59.5)	17 (40.5)		29 (69.0)	13 (31.0)	
Student	322 (66.1)	165 (33.9)		376 (77.2)	111 (22.8)		342 (70.2)	145 (29.8)	
Unemployed	96 (69.6)	42 (30.4)		110 (79.7)	28 (20.3)		109 (79.0)	29 (21.0)	
Others^a^	17 (56.7)	13 (43.3)		19 (63.3)	11 (36.7)		20 (66.7)	10 (33.3)	
Health care worker, n (%)									
Yes	71 (67.0)	35 (33.0)	0.508	77 (72.6)	29 (27.4)	0.870	106 (68.9)	33 (31.1)	0.899
No	553 (63.7)	315 (36.3)		637 (73.4)	231 (26.6)		603 (69.5)	265 (30.5)	
Income, n (%)									
≤30000 taka	321 (64.8)	174 (35.2)	0.460	362 (73.1)	133 (26.9)	0.651	339 (68.5)	156 (31.5)	0.827
>30000 taka	241 (62.4)	145 (37.6)		277 (71.8)	109 (28.2)		267 (69.2)	119 (30.8)	
Job Status, n (%)									
Full time job	112 (57.1)	84 (42.9)	0.043	126 (64.3)	70 (35.7)	0.008	125 (63.8)	71 (36.2)	0.201
Part time job	35 (66.0)	18 (34.0)		42 (79.2)	11 (20.8)		35 (68.0)	18 (34.0)	
Lost job during lockdown	45 (76.3)	14 (23.7)		48 (81.4)	11 (18.6)		44 (74.6)	15 (25.4)	
Not applicable	432 (64.9)	234 (35.1)		498 (74.8)	168 (25.2)		472 (70.9)	194 (29.1)	
Business status, n (%)									
Ongoing business	17 (54.8)	14 (45.2)	0.583	18 (58.1)	13 (41.9)	0.257	23 (74.2)	8 (25.8)	0.848
Business closed for now	42 (65.6)	22 (34.4)		47 (73.4)	17 (26.6)		42 (65.6)	22 (34.4)	
Lost investment	7 (77.8)	2 (22.2)		6 (66.7)	3 (33.3)		6 (66.7)	3 (33.3)	
Not applicable	558 (64.1)	312 (35.9)		643 (73.9)	227 (26.1)		676 (69.4)	298 (30.6)	
Source of information regarding COVID-19, n (%)									
Facebook	398 (63.5)	229 (36.5)	0.607	464 (74.0)	163 (26.0)	0.508	447 (71.3)	180 (28.7)	0.086
Other media	226 (65.1)	121 (34.9)		250 (72.0)	97 (28.0)		229 (66.0)	118 (34.0)	

*p*-value determined by Chi-square test.

Definitions: Anxiety- Present: GAD-7 ≥5, Absent: GAD-7 <5; Depression- Present: PHQ-9 ≥5, Absent: PHQ-9 <5; Stress- Present: PSS ≥14, Absent: PSS< 14.

a Other jobs include-intern, house tutor, day laborer, filmmaker, painter, freelancer, and self-employed physician.

Most of the participants had mild depression (n = 295, 30.3%), minimal anxiety (n = 350, 31.4%), and moderate stress (n = 657, 67.5%). However, severity of depression and anxiety was significantly higher among young people than adults (p < 0.001, and p = 0.015 respectively). Severity of stress was statistically similar across age groups (p = 0.623) (Table 3).

**Table 3:** Subtypes of depression, anxiety and stress among participants (n = 974).

Sub-category	Total (n = 974) n (%)	≤24 years (n = 473) n (%)	>24 years (n = 501) n (%)	p-value
**Depression (PHQ-9)**				
No depression (0–4)	260 (26.7)	103 (21.8)	157 (31.3)	<0.001
Mild depression (5–9)	295 (30.3)	134 (28.3)	161 (32.1)	
Moderate depression (10–14)	210 (21.6)	123 (26.0)	87 (17.4)	
Moderately severe depression (15–19)	148 (15.2)	79 (16.7)	69 (13.8)	
Severe depression (20–27)	61 (6.3)	34 (7.2)	27 (5.4)	
Mean PHQ-9 score [Mean (±SD)]	9.19 ± 6.15	9.95 ± 6.10	8.48 ± 6.12	<0.001
**Anxiety (GAD-7)**				
Minimal anxiety (0–4)	350 (35.9)	155 (32.8)	195 (38.9)	0.015
Mild anxiety (score 5–9)	306 (31.4)	154 (32.6)	152 (30.3)	
Moderate anxiety (10–14)	173 (17.8)	78 (16.5)	95 (19.0)	
Severe anxiety (15–21)	145 (14.9)	86 (18.2)	59 (11.8)	
Mean GAD-7 score [Mean (±SD)]	7.57 ± 5.61	7.98 ± 5.74	7.19 ± 5.47	0.027
**Stress (PSS)**				
Low stress (0–13)	298 (30.6)	138 (29.2)	160 (31.9)	0.623
Moderate stress (14–26)	657 (67.5)	325 (68.7)	332 (66.3)	
High perceived stress (27–40)	19 (2.0)	10 (2.1)	9 (1.8)	
Mean Perceived Stress Scale (PSS) score [Mean (±SD)]	16.02 ± 5.55	16.32 ± 5.52	15.74 ± 5.57	0.104

In the univariable logistic regression analysis, age ≤24 years, female sex and unemployment were associated with anxiety. The young adults (age ≤24 years) were 1.307 times more likely (95% Confidence Interval [CI] 1.005–1.701), females were more than 2.171 times more likely (95% CI: 1.548–3.044) and the unemployed individuals were 1.567 times more likely (95% CI: 1.196–2.053) to have anxiety than the adults, male and employed individuals, respectively. On multivariate analysis, no independent variable could make a unique statistical significance to the model regarding anxiety. Again, on univariate regression age ≤24 years, female sex, unemployment and being single were significant contributors to depression. But unemployment was found to be the only significant independent predictors of depression in the study after multivariable regression (Adjusted Odds Ratio [AOR] 3.642; CI: 1.005–13.2). After multivariable analysis perceived stress was significantly associated with female sex (AOR 1.638; CI: 1.158–2.317) and unemployment (AOR 1.399; CI:1.055–1.855) (see Table 4).

**Table 4:** Predictors of anxiety, depression and stress among participants (n = 974).

Variable	Reference Category	Anxiety		Depression		Stress	
		Univariable OR (95% CI)	Multivariable AOR (95% CI)	Univariable OR (95% CI)	Multivariable AOR (95% CI)	Univariable OR (95% CI)	Multivariable AOR (95% CI)
Age ≤24 years	>24 years	1.307 (1.005–1.701)∗	1.177 (0.602–2.298)	1.639 (1.228–2.188)‡	1.036 (0.479–2.239)	1.139 (0.867–1.497)	
Sex (Female)	Male	2.171 (1.548–3.044)†	1.684 (0.831–3.414)	2.009 (1.380–2.924)†	2.022 (0.915–4.468)	1.704 (1.207–2.405)∗	1.638 (1.158–2.317)∗
Occupation (Unemployed)	Employed	1.567 (1.196–2.053)‡	2.436 (0.895–6.632)	2.121 (1.587–2.835)†	3.642 (1.005–13.200)∗	1.459 (1.102–1.930)∗	1.399 (1.055–1.855)∗
Marital status (Married)	Single	0.820 (0.617–1.008)		0.598 (0.443–0.807)‡	0.640 (0.371–1.105)	0.780 (0.582–1.045)	
Residence (Urban)	Rural	1.123 (0.825–1.530)		1.310 (0.944–1.818)		1.096 (0.795–1.511)	
Education (graduate& above)	Under HSC	0.910 (.676–1.224)		0.739 (0.530–1.031)		1.125 (0.830–1.525)	
Health care worker	No	1.156 (0.753–1.772)		0.963 (0.612–1.514)		0.972 (0.629–1.503)	
Income (≤300000 taka)	>30,000 taka	1.110 (0.842–1.464)		1.071 (0.795–1.442)		0.969 (0.727–1.291)	
Job status (No current job)	Having job	2.230 (1.163–4.276)∗	0.976 (0.324–2.935)	2.104 (1.038–4.266)∗	0.582 (0.146–2.318)	1.632 (0.860–3.097)	
Business Status (No business)	Having business	1.681 (0.712–3.972)		1.914 (0.794–4.612)		0.668 (0.261–1.707)	

∗ Statistical significance at *p* < 0.05.

† Statistical significance at *p* < 0.001.

‡ Statistical significance at *p* = 0.001.

## 4 Discussion

The COVID-19 pandemic has provoked a worldwide emergency and havocked the day-to-day lives of the general population. Countries around the world are going through a challenging situation as the number of infected patients is increasing daily. Like so, the government of Bangladesh implemented countrywide lockdowns at the initial stage of the pandemic to prevent any further spread of the virus. However, the fear of contracting the virus on the one hand and an apprehension of economic uncertainty on the other riddled with ‘infodemic’ from social media led to a mixed range of psychological and emotional reactions among the general population. Therefore, we aimed to assess the prevalence and predictors of anxiety, depression and stress among the adults not diagnosed with COVID-19 in Bangladesh amidst the pandemic.

In our study most of the participants were aged ≤24 years with minimum age being 16 years. According to United Nations secretariate people with an age 15–24 years are considered youth or young people [24]. Therefore, we focused on the mental health status of young in the middle of COVID-19 pandemic with a comparison to adults who were predominantly at their early middle age.

We found that 64.1% of the participants had anxiety (mild to severe), 73.3% had depression (mild to severe) and 69.4% had stress (moderate to high). Our findings are higher than those found by Mamun et al [14] and Banna et al [15] during the early and late parts of the first month (April 2020), respectively, of lockdown among the adult population of Bangladesh. Mamun and colleagues found depressive symptoms among 33.3% of participants (measured by the Bangla PHQ), while Banna and colleagues reported anxiety, depression and stress symptoms in 33.7%, 57.9% and 59.7% of adults, respectively (measured by the DASS-21). Our study was conducted after the first month of lockdown, indicating a slow rise in the mental health impacts of COVID-19 and associated measures at the second month (May 2020). However, the study by Zubayer et al [25] during the third month of lockdown (June 2020) found anxiety, depression and stress in 47.2%, 46.0% and 32.5% of the adult population, respectively (measured by the DASS-21). These seem to indicate an adaptation of people with the novel situation over time. However, the differences among these studies might be due to differences in instruments used and participants selected.

We also noted that the youth were significantly more affected by anxiety and depression than adults which is the supported by the findings of Banna et al [15] who reported that prevalence of anxiety and depression was high among those aged ≤23 years compared older participants. Interestingly a study conducted among job-seeking young graduates (mean age 24.12 ± 1.55 years) of Bangladesh in 2018 [26] found that 53.2%, 49.6% and 26.4% of them had anxiety, depression and stress, respectively. While we found 67.2%, 78.2% and 70.8% of our youth participants (≤24 years) had anxiety, depression and stress, respectively. This clearly indicates an increase in the mental health problems during the COVID-19 pandemic associated lockdowns which might have been precipitated by the uncertainty of earning amidst an environment where people were already loosing jobs and business.

Our study was conducted only among individuals not diagnosed with COVID-19. In comparison, the first report on COVID-19 patients by Hasan et al [27] found anxiety and depression among 60% and 52.9% of participants, respectively. This is similar to that of people without COVID-19 infection found in our study and indicates a comparable impact of the pandemic in people irrespective of their infection status. Social and electronic media exposure might have been an important contributor to perceived anxiety, as evidenced by Hossain and colleagues [28]. Our finding that more than half of the participants searched and/or found their information regarding COVID-19 from social media endorses this assumption. However, the overall proportion of people suffering from anxiety, depression, and stress symptoms in our country seems to be higher than that found in developed countries [29].

In the past, during other outbreaks, such as ‘Ebola’ or ‘SARS’, individuals and communities at the national and international levels had a wide spectrum of psychosocial consequences due to the sudden outbreak of the disease. It is likely that people were afraid of falling sick, being helpless, hopeless and stigmatized and even dying. Constant support of mental and psychosocial well-being in different groups during the pandemic should be the highest priority in such situations [30, 31]. The provision of government support for the general population to the highest extent during the lockdown lessened the mental health consequences in developed countries.

Demographic variables showed that anxiety, depression and stress were more prevalent in young people. On univariate analysis, depression was found to be significantly more common among people youth aged ≤24 years (OR 1.639; CI: 1.228–2.188), female sex (OR 2.009; CI: 1.380–2.924), and unemployed (OR 2.121, CI: 1.587–2.835). One study conducted among youth in the Middle East [32] found that being female, being in quarantine for two weeks, and increased use of the internet were important determinants of stress, anxiety and depression. The reason might as well fit in our youths.

Similar to the Chinese community [30], we found that females had a greater psychological impact on the COVID-19 outbreak than their male counterparts. Similarly, Hamadani et al [33] found that COVID-19 lockdowns caused significant economic, psychosocial, and physical risks to the wellbeing of women in Bangladesh. Women, in general, are at a higher risk for psychological events and report more severe symptoms of depression, anxiety, and distress [34]. Social, cultural and existing gender norms tend to make women relatively more vulnerable than men to mental health disorders.

Unemployed participants were significantly more likely to be anxious, depressed, and stressed than employed participants, and unemployment was the single most important factor for depression, and stress in the current study. We found that a considerable percentage of people lost their jobs (6.1%) and lost investments (0.9%) during this pandemic. Hamadani and colleagues [31] noted a sizable reduction in median family income in rural areas. All of these results indicate that people, particularly those who were not infected by COVID-19, were influenced in a negative way by the socioeconomic consequences of the lockdown.

### 4.1 Limitations

The major limitation of the study was that it represented a relatively young population using Facebook, thus making the results non-generalizable to the adult population of the country. Randomization of participants was not possible either.

## 5 Conclusion

We found a high proportion of anxiety, depression and stress symptoms among the young people not diagnosed with COVID-19 infection during the pandemic associated lockdown in Bangladesh. Unemployment was one of the single most notable predictors for depression and stress. Stress was significantly more common in females than in males. The findings warrant further monitoring of the Bangladeshi youth's mental health as the pandemic continues, and we await vaccination programs to be successful soon to root out the COVID-19 pandemic.

## Declarations

### Author contribution statement

Md. Abdullah Saeed Khan, Mohammad Jahid Hasan, Sourav Debnath: Conceived and designed the experiments; Performed the experiments; Analyzed and interpreted the data; Wrote the paper.

Mohammad Sorowar Hossain, Monjur Rahman: Conceived and designed the experiments; Performed the experiments; Analyzed and interpreted the data; Contributed reagents, materials, analysis tools or data.

Md. Shahnoor Islam: Conceived and designed the experiments; Performed the experiments; Analyzed and interpreted the data; Contributed reagents, materials, analysis tools or data; Wrote the paper.

Susmita Zaman, Noor-E-Ambia: Performed the experiments; Analyzed and interpreted the data; Contributed reagents, materials, analysis tools or data.

Anindita Das Barshan, Tamanna Tabassum: Performed the experiments; Analyzed and interpreted the data; Contributed reagents, materials, analysis tools or data; Wrote the paper.

### Funding statement

This research did not receive any specific grant from funding agencies in the public, commercial, or not-for-profit sectors.

### Data availability statement

Data will be made available on request.

### Declaration of interests statement

The authors declare no conflict of interest.

### Additional information

No additional information is available for this paper.
